# Inter-Beam Co-Channel Downlink and Uplink Interference for 5G New Radio in mm-Wave Bands ^†^

**DOI:** 10.3390/s21030793

**Published:** 2021-01-25

**Authors:** Kamil Bechta, Jan M. Kelner, Cezary Ziółkowski, Leszek Nowosielski

**Affiliations:** 1 Nokia Solutions and Networks, 54-130 Wrocław, Poland; kamil.bechta@nokia.com; 2 Institute of Communications Systems, Faculty of Electronics, Military University of Technology, 00-908 Warsaw, Poland; cezary.ziolkowski@wat.edu.pl (C.Z.); leszek.nowosielski@wat.edu.pl (L.N.)

**Keywords:** wireless mobile communications, 5G, millimeter-wave, multi-beam antenna system, wireless downlink and uplink, co-channel interference, signal-to-interference ratio, multi-ellipsoidal propagation model, simulation studies

## Abstract

This paper presents a methodology for assessing co-channel interference that arises in multi-beam transmitting and receiving antennas used in fifth-generation (5G) systems. This evaluation is essential for minimizing spectral resources, which allows for using the same frequency bands in angularly separated antenna beams of a 5G-based station (gNodeB). In the developed methodology, a multi-ellipsoidal propagation model (MPM) provides a mapping of the multipath propagation phenomenon and considers the directivity of antenna beams. To demonstrate the designation procedure of interference level we use simulation tests. For exemplary scenarios in downlink and uplink, we showed changes in a signal-to-interference ratio versus a separation angle between the serving (useful) and interfering beams and the distance between the gNodeB and user equipment. This evaluation is the basis for determining the minimum separation angle for which an acceptable interference level is ensured. The analysis was carried out for the lower millimeter-wave band, which is planned to use in 5G micro-cells base stations.

## 1. Introduction

This paper focuses on a methodology for assessing co-channel interference occurring in fifth-generation (5G) networks, in which a directional wireless link is one of the key techniques [1,2]. To this aim, especially in frequency bands below 6 GHz, 5G new radio (NR) base stations (gNodeBs) use multi-beam antenna systems based on massive multiple-input-multiple-output (massive-MIMO) technology, which is enabled by digital beamforming [3,4]. This solution reduces the energy expenditure due to the high gain and narrow width of the beams. The needed increase in the radio link capacity is obtained thanks to an energy balance improvement. On the other hand, massive-MIMO ensures the efficient use of spectral resources. An appropriate angular separation between individual beams makes it possible to use the same frequency channels. However, the minimization of spectral resources offered by this technology is associated with the need to assess the interplay of signals received by individual beams. In the millimeter-waves (mmWaves) frequency range, where early implementations of 5G are based on analog beamforming, the multi-beam transmissions inside a single cell sector are obtained by multiple sub-arrays, which constitute the full antenna array. Each of these sub-arrays is connected to individual transmission–reception chain and enables the simultaneous generation of multiple beams. Hitherto, evaluation methodologies of co-channel interference mainly concerned omnidirectional and sectoral antennas and homogeneous environments, e.g., [5,6,7]. From the viewpoint of 5G systems that use mmWaves, massive-MIMO, and beamforming, such analysis should consider narrow beams of antenna systems. Examples of such studies are presented in [8,9,10,11]. On the other hand, the analysis of co-channel interference is essential to assessing the coexistence and compatible functioning of different radio systems. The importance of this problem is presented in [9]. Besides, many new procedures in massive-MIMO and beamforming systems that increase the efficiency of 5G require assessing the level of interference between the antenna beams to and from individual users. The partial-nulling based statistical beamforming is an example of such a procedure, the use of which is based on the division of all users into two groups with a significantly different degree of spatial correlation [12]. The solution presented in [13] is another example that increases the spectral efficiency in massive-MIMO systems. These selected examples show the importance of assessing the level of interference between the beams of the antenna system from other users in developing and implementing new solutions.

The utilization of narrow beams and the dominance of the multipath propagation phenomenon in urban areas significantly change interference analysis methods. In this case, the practical used method of the interference assessment is based on simulation tests. Parameters of transmitted and received signals as well as their statistical properties for various types of propagation environments are input for these studies. The 3rd Generation Partnership Project (3GPP) standard [14] is commonly used for this aim. This approach recommends using deterministic cluster delay lines (CDLs) for link-level simulations, where average angles of departure (AODs) and arrival (AOAs), in addition to tapped delay line (TDL), are defined. For system-level simulations, where a statistical approach is more appropriate, the 3GPP standard [14] recommends the full three-dimensional (3D) modeling of a radio channel.

The interference topic is widely represented in the literature. On the one hand, there are works on counteracting interference in the emerging and future systems. Examples include software algorithms and hardware solutions aimed at interference cancellation [15], mitigation [16], or awareness [17,18]. In this case, the currently proposed solutions are mainly dedicated to multi-antenna systems. On the other hand, papers focusing on the interference evaluation and measurement [19] methodologies are presented. In general, the interference analysis can be performed at any distance from the signal source antenna. In the case of a near-field, the influence between the individual elements of the multi-antenna system can be investigated [20,21,22,23,24]. In the case of a far-field, inter-beam [25,26], inter-cell [18,26,27], or inter-system interferences, i.e., the coexistence of different systems and networks [9,11,28], might be studied. In the literature, the vast majority of scientific works concern coexistence topic and the inter-cell interference assessment, rather than inter-beam interference.

In this paper, we present a methodology for assessing co-channel interference that is resulting from the utilization of the same frequency channels in different beams of the gNodeB antenna system. To evaluate the signal-to-interference ratio (SIR), we use simulation tests that are based on a 3D multi-ellipsoidal propagation model (MPM) [29,30,31]. However, the proposed methodology differs from the simulation approach recommended by the 3GPP standard for link-level evaluations. In our methodology, using the MPM as a geometry-based model (GBM) provides a statistical SIR metric in contrast to the 3GPP approach with the pre-determined AODs and AOAs. In our solution, the knowledge of spatial parameters such as the average AODs and AOAs of propagation paths is not required to obtain results and the use of any power delay profile (PDP) or the TDL makes it possible to adapt this model to any propagation scenarios. The use of the MPM and antenna radiation patterns allow determining a power angular spectrum (PAS) of the received signals. The obtained PASs are the basis for the SIR assessment in the multi-beam antenna system. The MPM geometry is constructed based on the PDP or TDL, which describe the transmission properties of a propagation environment. This original way of mapping the effects of propagation phenomena enables obtaining a fully statistical SIR evaluation. It ensures a relationship of simulation results with the analyzed type of propagation environment. The presented analysis is an extension of the paper [32], which focused only on the downlink (DL) interference in multi-beam 5G macro-cell for 3.5 GHz band and under non-line-of-sight (NLOS) conditions. In that case, we used a two-dimensional multi-elliptical model [31]. In this study, based on the 3D MPM, we discuss the inter-beam co-channel interference in DL and additionally uplink (UL) for 28 GHz band, under line-of-sight (LOS) and NLOS conditions. Using the 3D MPM to modeling the inter-beam interference level testifies to the novelty of the presented approach. In this case, an original approach to the UL interference assessment was proposed, taking path loss corrections into account, compared to the DL scenario [32] shown previously.

The mutual configuration of the transmitting and receiving antenna beams is not only the factor considered in our methodology. The presented solution of the SIR determination also provides a mapping of the propagation environment influence on the PAS of the received signals. The 3GPP methodology also takes this impact into account. However, it is limited to well-defined types of environments that are defined based on the determined channel characteristics and distributions of channel parameters. In the case of the MPM methodology presented in this paper, we have the option to assess the SIR for any type of propagation environment, whose transmission properties are defined by an appropriate PDP. This fact significantly distinguishes the developed method and proves its originality.

The remainder of the paper is organized as follows: Section 2 shows the construction way of the MPM geometry based on the transmission properties of the propagation environment, i.e., the TDL. The essence of the PAS determination procedure and then the SIR assessment is presented in Section 3. In Section 4 and Section 5, the description of the analyzed scenarios for the DL and UL transmissions and the results of simulation studies are drawn. Section 6 contains conclusions.

## 2. Multi-Ellipsoidal Propagation Model

**Figure 1:**
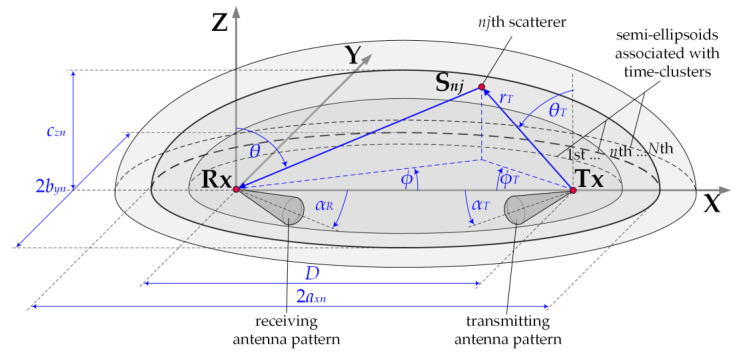
MPM geometrical structure considering Earth surface.

In 5G networks, designing wireless links with narrow beams and beamforming technology enforces the use of GBMs. It is particularly important in relation to urbanized areas. In this case, there is a large directional variation in the received powers. The use of GBMs ensures the spatial power distribution in the vicinity of the receiving antenna. In combination with the narrow beam patterns, this approach gives the possibility of a statistical evaluation of the transmission properties of the wireless link. The MPM is one of the GBMs. The set of confocal ellipsoids forms its geometrical structure representing the potential locations of the scattering elements for an emitted radio wave. In the foci of the ellipsoids, a transmitter (Tx) and receiver (Rx) are located. In propagation scenarios, where the Tx and Rx are on the Earth’s surface, the MPM structure is represented by a set of semi-ellipsoids, as shown in Figure 1 [31].

The MPM geometrical structure is closely related to the transmission properties of the propagation environment, which may be described by the PDP or TDL. In multipath environments, we can observe the occurrence of several or a dozen taps or local extremes in the TDLs or PDPs, respectively. It means that as a result of scattering on terrain obstacles, the electromagnetic wave reaches the Rx by different propagation paths but with the same delays $\tau_{n}$ for n=1,2,…,N [33]. Thus, based on the properties of geometrical figures, the most probable locations of the scattering elements form an ellipsoid. Of course, the number of ellipsoids is equal to the number N of time-clusters representing the taps or local extremes in the TDL or PDP, respectively. If the distance between the Tx and Rx is equal to D, then the parameters of individual ellipsoids such as a major, *a_xn_*, and minor semi-axes *b_yn_*, *c_zn_* describe the following relationships:

(1) $$a_{xn}=\frac{1}{2}\left(c\tau_{n}+D\right),$$

(2) $$b_{yn}=c_{zn}=\frac{1}{2}\sqrt{c\tau_{n}\left(c\tau_{n}+2D\right)},$$

where *c* denotes the speed of light.

The geometrical structure of the MPM is described in detail in [29,30,31]. The 3D MPM may be reduced to the 2D multi-elliptical model, where propagation phenomena in the azimuth plane are dominant [31]. For this modeling procedure in relation to other GBMs, minimizing the estimation error of the PAS is shown in [34]. In the MPM, the phenomenon of local scattering around the transmitting and receiving antennas is also included. In this case, the AOAs of propagation paths are generated based on the 2D von Mises distribution [29,35]:

(3) $$\begin{array}{c} f_{0}\left(\theta ,\phi \right)=C_{0}\frac{\exp\left(\gamma_{\theta }\cos\left(90{}^{\circ}-\theta \right)\right)}{2\pi I_{0}\left(\gamma_{\theta }\right)}\cdot \frac{\exp\left(\gamma_{\phi }\cos\phi \right)}{2\pi I_{0}\left(\gamma_{\phi }\right)}\\ \mathrm{for}\;\theta \in \langle 0,90{}^{\circ})\;\mathrm{and}\;\phi \in \langle -180{}^{\circ},180{}^{\circ}), \end{array}$$

where (θ,ϕ) is AOA in the elevation and azimuth planes, respectively, $\gamma_{\theta }$ and $\gamma_{\phi }$ define the angular dispersion of the components in the elevation and azimuth planes, respectively, $I_{0}\left(\cdot \right)$ is the zero-order modified Bessel function of the imaginary argument, and $C_{0}$ represents the normalizing constant such that $\frac{C_{0}}{2\pi I_{0}\left(\gamma_{\theta }\right)}\overset{90{}^{\circ}}{\underset{0}{\int }}\exp\left(\gamma_{\theta }\cos\left(90{}^{\circ}-\theta \right)\right)d\theta =1$.

## 3. Evaluation of Co-Channel Interference in Multi-Beam Antenna

The co-channel interference assessment is based on the SIR measure defined as:

(4) $$\begin{array}{ccc} SIR=\frac{P_{S}}{P_{I}}\left(\frac{W}{W}\right) & \overset{}{\underset{}{↔}} & SIR\left(\mathrm{dB}\right)=P_{S}\left(\mathrm{dBm}\right)-P_{I} \end{array}\left(\mathrm{dBm}\right),$$

where $P_{S}$ and $P_{I}$ are the powers of the serving and interfering signal, respectively, which occur at the output of the receiving antenna. In the multi-beam receiving antenna, the interference signal is from a wireless link whose receiving antenna beam is formed in the same frequency band as the serving beam. From the SIR definition, it follows that the main problem of assessing this measure relies on determining $P_{S}$ and $P_{I}.$ Note that these powers can be calculated based on the appropriate PASs, $p_{S,I}\left(\theta ,\phi \right)$, which are seen at the output of the receiving antenna, namely:

(5) $$P_{S,I}=\underset{\left(\theta ,\phi \right)}{\iint }p_{S,I}\left(\theta ,\phi \right)d\theta d\phi .$$

However, these distributions depend on the power pattern of the serving beam [30]:

(6) $$p_{S,I}\left(\theta ,f\right)={\tilde{p}}_{S,I}\left(\theta ,f\right){\left|g\left(\theta ,f\right)\right|}^{2},$$

where ${\tilde{p}}_{S,I}\left(\theta ,\phi \right)$ represent the PASs in the vicinity of the receiving antenna and ${\left|g\left(\theta ,\phi \right)\right|}^{2}$ is the normalized power pattern of the receiving antenna.

Hence, it follows that the problem of the SIR assessment boils down to determining ${\tilde{p}}_{S,I}\left(\theta ,\phi \right)$. The developed methodology uses simulation tests to determine these PASs. The input data for simulation procedures that condition the estimation of ${\tilde{p}}_{S,I}\left(\theta ,\phi \right)$ is a set of the following parameters and characteristics:

- normalized power patterns ${\left|g_{S}\left(\theta_{T},\phi_{T}\right)\right|}^{2}$, ${\left|g_{I}\left(\theta_{T},\phi_{T}\right)\right|}^{2}$, and ${\left|g\left(\theta ,\phi \right)\right|}^{2}$, of the serving and interfering transmitting and receiving beams, respectively, where $\left(\theta_{T},\phi_{T}\right)$ denotes AOD in the elevation and azimuth planes, respectively;
- gains $G_{S}$, $G_{I}$, and G of the serving and interfering transmitting and receiving beams, respectively;
- the Tx-Rx distances, i.e., $D_{S}$ and $D_{I}$ between the serving and interfering mobile stations (user equipment, i.e., UE-S and UE-I) and gNodeB for the UL scenario, respectively, or $D_{S}=D_{I}=D$ for the DL scenario;
- the type of propagation environment defined by the TDL or PDP and rms delay spread.
- Estimation of ${\tilde{p}}_{S,I}\left(\theta ,\phi \right)$ consists in the generation of a set of propagation paths departing from the transmitting antennas of the serving and interfering links and their transformation in a system composed of the semi-ellipsoid set. The generation procedure of AODs, $\left(\theta_{T},\phi_{T}\right),$ uses the properties of the normalized power radiation patterns [36]:
  (7) $$\frac{1}{4\pi }\underset{\left(\theta_{T},\phi_{T}\right)}{\iint }{\left|g_{S,I}\left(\theta_{T},\phi_{T}\right)\right|}^{2}\sin\theta_{T}d\theta_{T}d\phi_{T}=1.$$

The function under integral is non-negative. Therefore, the normalized power radiation patterns meet the axioms of a probability density function. Hence, we can express the distribution of AOD as [36]:

(8) $$f_{S,I}\left(\theta_{T},\phi_{T}\right)=\frac{1}{4\pi }{\left|g_{S,I}\left(\theta_{T},\phi_{T}\right)\right|}^{2}\sin\theta_{T}.$$

The geometry structure of the MPM represents the statistical locations of the scattering elements. Thus, the intersection of the radiated path with individual semi-ellipses indicates the scattering places of this path. Knowing the AODs, $\left(\theta_{T},\phi_{T}\right),$ of radiated propagation paths, we can determine for each of them the radial coordinate $r_{T}$ in a spherical system with an origin in the Tx (UE-S or UE-I). For the selected time-cluster (ellipsoid), this coordinate is described by [29]:

(9) $$r_{T}=-\frac{1}{2a}b_{y}^{2}D\sin\theta_{T}\cos\phi_{T}+\frac{1}{2a}\sqrt{{\left(b_{y}^{2}D\sin\theta_{T}\cos\phi_{T}\right)}^{2}+4ab_{y}^{2}\left(a_{x}^{2}-\frac{D^{2}}{4}\right)},$$

where $a={\left(b_{y}\sin\theta_{T}\cos\phi_{T}\right)}^{2}+a_{x}^{2}\left(\cos^{2}\theta_{T}+{\left(\sin\theta_{T}\sin\phi_{T}\right)}^{2}\right)$.

Appropriate coordinate transformation resulting from translation the system origin to the Rx allows determining AOA, (θ,ϕ), for propagation paths reaching the Rx [29]:

(10) $$\theta =\arctan\frac{\sqrt{{\left(r_{T}\sin\theta_{T}\cos\phi_{T}+D\right)}^{2}+{\left(r_{T}\sin\theta_{T}\sin\phi_{T}\right)}^{2}}}{r_{T}\cos\theta_{T}},$$

(11) $$\phi =\arctan\frac{r_{T}\sin\theta_{T}\sin\phi_{T}}{r_{T}\sin\theta_{T}\cos\phi_{T}+D}.$$

In addition to the AOAs of propagation paths reaching the Rx with delays, the local scattering paths are also included. In this case, the AOAs are generated using the von Mises distribution described by Equation (3).

Powers $\tilde{p}$ of individual paths are determined based on the PDP or TDL. To generate these powers, we use exponential distributions, $f\left(\tilde{p}\right),$ whose parameters (i.e., mean values $p_{n}$ for n=1,2,…,N) are equal to powers of the taps or local extremes occurring in the TDL or PDP, respectively:

(12) $$f\left(\tilde{p}\right)=\{\begin{array}{lll} \frac{1}{p_{n}}\exp\left(\frac{\tilde{p}}{p_{n}}\right) & \mathrm{for} & \tilde{p}\geq 0,\\ 0 & \mathrm{for} & \tilde{p}<0, \end{array}$$

where $p_{n}$ is the *n*th local extreme of the PDP (or *n*th tap value of the TDL), which corresponds to the propagation paths reaching from the *n*th semi-ellipsoid, i.e., with the delay $\tau_{n}$.

As a simulation result, we get the set of $\left\{\left(\theta ,\phi ,\tilde{p}\right)\right\}$ that enables estimating ${\tilde{p}}_{S,I}\left(\theta ,\phi \right)$ [29]. Additional multiplication of each $\tilde{p}$ value by the appropriate value of ${\left|g\left(\theta ,\phi \right)\right|}^{2}$ gives us the set of {(θ,ϕ,p)}, which is the basis for estimating $p_{S,I}\left(\theta ,\phi \right)$. A detailed description of the practical implementation of the estimation procedure is provided in [30]. As a result, we can determine $P_{S}$ and $P_{I}$ based on Equation (5).

For the DL scenario, the SIR may be calculated based on Equation (4), because the serving and interfering beams are generated by the same gNodeB, i.e., $D_{S}=D_{I}=D$. However, for the UL scenario, the SIR assessment requires additional consideration of attenuation resulted from a difference in the distances between the gNodeB and UE-S or UE-I. Considering this fact, we have:

(13) $$\begin{array}{ccc} SIR=\frac{P_{S}}{P_{I}}\Delta PL & \overset{}{\underset{}{↔}} & SIR\left(\mathrm{dB}\right)=P_{S}\left(\mathrm{dBm}\right)-P_{I} \end{array}\left(\mathrm{dBm}\right)+\Delta PL\left(\mathrm{dB}\right)$$

where:

(14) $$\begin{array}{ccc} \Delta PL=\frac{PL\left(D_{S}\right)}{PL\left(D_{I}\right)} & \overset{}{\underset{}{↔}} & \Delta PL\left(\mathrm{dB}\right)=PL\left(D_{S}\right)\left(\mathrm{dB}\right)-PL\left(D_{I}\right) \end{array}\left(\mathrm{dB}\right)$$

represents the relationship between attenuation of propagation environment for different distances, $PL\left(D_{S}\right)$ and $PL\left(D_{I}\right)$ are path losses for the wireless links between the UE-S or UE-I and gNodeB, respectively. For assessing ΔPL, we use a close-in free space reference distance path loss model presented in [37]. To take the influence of variable weather conditions into account that are related to atmospheric precipitation, the used path loss model should be corrected based on ITU-R recommendations [38,39] or other approaches proposed in the literature, e.g., [40,41].

Generally, the proposed methodology of the interference evaluation consists of the following stages:

- defining the scenario parameters,
- determining the MPM parameters,
- determining the PASs for the serving and interfering links based on simulation studies,
- calculating the powers for the determined PASs,
- calculating the SIR finally.

## 4. Assumptions and Scenarios of Simulation Studies

**Figure 2:**
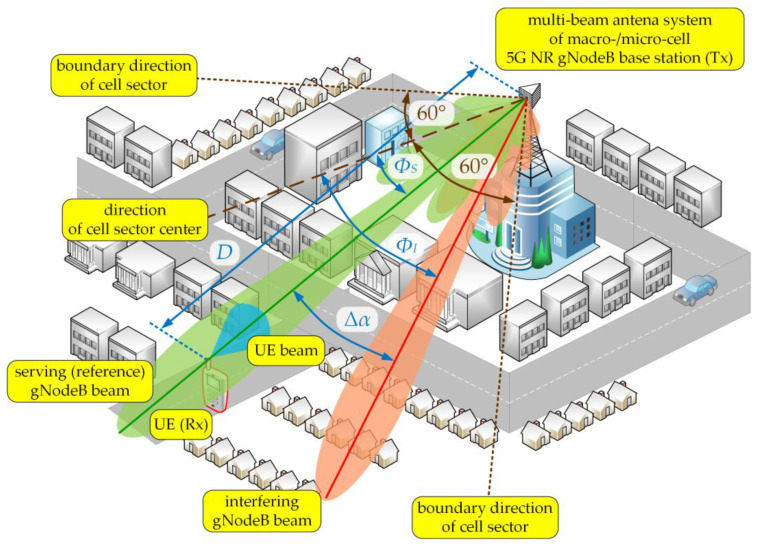
DL spatial scenario of simulation studies [32].

**Figure 3:**
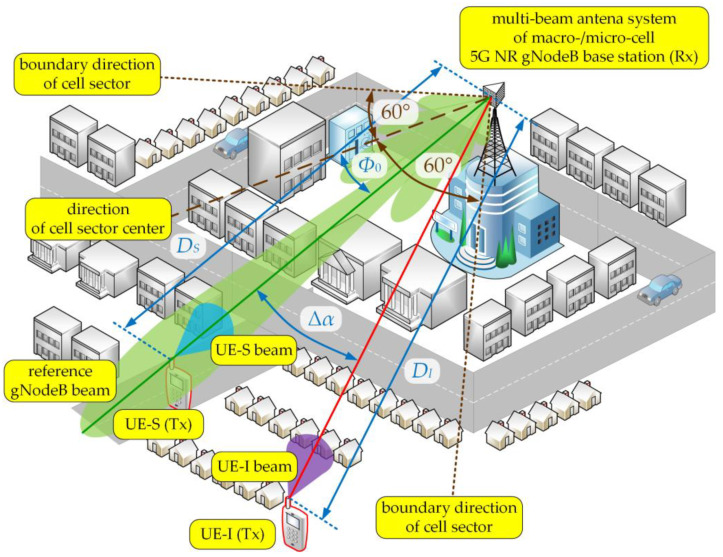
UL spatial scenario of simulation studies.

The aim of the simulation tests is to present a method of modeling and assessing the co-channel interference that arises in the radio link with a multi-beam antenna system. The studies focused on determining the SIR relationship on the separation angle Δα and changes in the distance D (or distances $D_{S}$ and $D_{I}$) between the UEs and gNodeB. The simulations were carried out for carrier frequency of 28 GHz, typical for the 5G micro- and pico-cells, where multiple sub-arrays and beamforming technologies are planned for implementation. Besides, we considered two scenarios for the DL and UL, which are illustrated in Figure 2 and Figure 3, respectively.

In the DL scenario, we assumed that the gNodeB was generating two beams (serving and interfering) in the selected sector that were operating in the same sub-band (frequency channel). Thus, the SIR assessment came down to determining $P_{S}$ and $P_{I}$ powers induced in the UE antenna that come from the signals generated by the serving and interfering beams of the gNodeB, respectively. The distances between the gNodeB (Tx) and UE (Rx) was equal to D. Besides, the serving (reference) gNodeB and UE beams were aligned, i.e., directed to each other ($\alpha_{TS}=0$ and $\alpha_{R}=0$, see Figure 1). In relation to the direction of the cell sector center, the reference and interfering gNodeB beams are oriented in $\Phi_{S}$ and $\Phi_{I}$ directions, respectively (see Figure 2). Thus, the separation angle of the beams is defined as:

(15) $$\Delta \alpha =\Phi_{S}-\Phi_{I},$$

Then the interfering beam orientation in relation to the Tx-Rx direction was equal to $\alpha_{TI}=\Delta \alpha$.

A similar scenario was taken into account for the UL transmission depicted in Figure 3. In this case, the analyzed gNodeB beam served one subscriber (UE-S) in its area, while another subscriber (UE-I) generated interferences towards this gNodeB beam. The UE-S and UE-I beams (Tx) were oriented to the gNodeB (Rx), i.e., $\alpha_{TS}=\alpha_{TI}=0$ (see Figure 1). Whereas the gNodeB beam directions to the UE-S and UE-I were equal to $\alpha_{RS}=0$ and $\alpha_{RI}=\Delta \alpha$, respectively. So, in both scenarios, the separation angle, Δα, was always related with the direction of the gNodeB beam for the interfering link. In relation to the direction of the cell sector center, the gNodeB beam direction was equal to $\Phi_{0}$ (see Figure 3). The distances between the gNodeB (Rx) and UE-S or UE-I (Tx) were equal to $D_{S}$ or $D_{I}$, respectively.

In our tests, the direction of the reference gNodeB beam overlapped with the cell sector center, i.e., $\Phi_{0}=\Phi_{S}=0$. Hence, we considered the change in separation angle in the ranges of 0°÷60°. When analyzing the SIR changes in relation to the beam separation angle, we considered discrete distance values between the gNodeB and UE (or UE-I in the UL scenario), i.e., $D_{S}=100\;m$ and $D=D_{I}\in \left\{50,100,150\right\}\;\left(m\right).$ In this case, Δα was changed from 0 to 60°, which corresponds to half of a 120° sector. Analyzing the SIR versus D or $D_{I}$ (for the DL or UL scenarios, respectively), we considered a continuous change of the distance in the ranges of 10÷250 m, whereas the separation angle has discrete values 15°, 20°, and 30°. For the UL scenario, we additionally used the close-in free space reference distance propagation models with path loss exponents equal to 1.9 and 4.5 for LOS and NLOS conditions, respectively [37].

**Figure 4:**
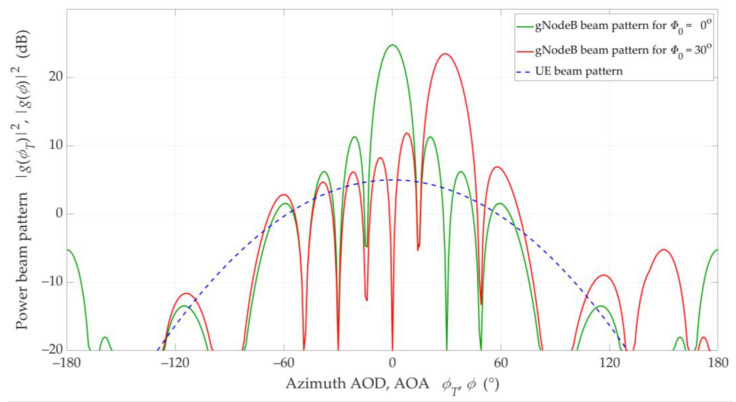
Antenna beam patterns of 1 × 1 UE and 12 × 8 gNodeB for *Φ*_0_ = 0° and *Φ*_0_ = 30° [32].

To model the antenna power radiation patterns, we adopted 3GPP recommendations [42]. Half-power beamwidths of main-lobes of the antenna beams were 90° for the UE and about 12° for the gNodeB, respectively. Single antenna beam patterns of the UE and gNodeB for direction $\Phi_{0}=0{}^{\circ}$ and $\Phi_{0}=30{}^{\circ}$ are illustrated in Figure 4 [32]. In the gNodeB, we used a vertical patch as an antenna array with a size 12 × 8 of elements, whereas the UE antenna consists of a single element.

**Figure 5:**
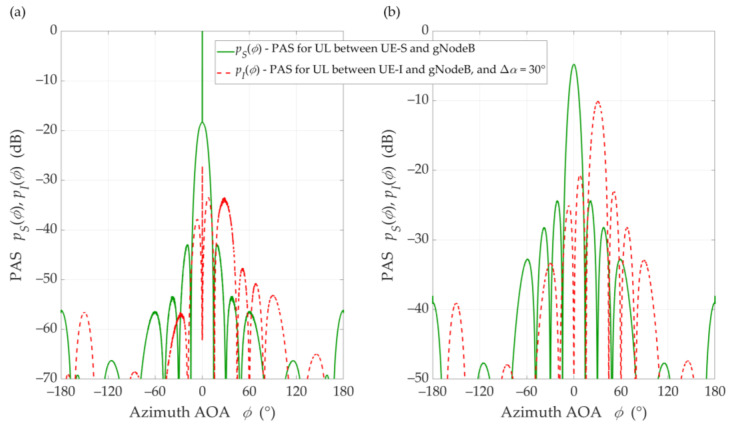
PASs of UE-S and UE-I in azimuth plane for ∆*α* = 30°, *D_S_* = *D_I_* = 100 m, under (**a**) LOS (TDL-D) and (**b**) NLOS (TDL-B) conditions.

The simulations were carried out for an urban macro (UMa) environment that is characterized by a normal delay profile with the rms delay spread equal to 266 ns [14]. To model the channel transmission properties, we adopted the TDLs with the 3GPP standard [14], i.e., TDL-D and TDL-B for LOS and NLOS conditions, respectively. To estimate $p_{S,I}\left(\theta ,\phi \right)$, we used the averaging PASs obtained in 3600 Monte-Carlo simulations. In each Monte-Carlo run, the PAS was obtained based on the generation of 10 random propagation paths for each time-cluster (ellipsoid). Figure 5 presents averaged PAS examples of the UE-S and UE-I in the azimuth plane for the UL scenario, $D_{S}=D_{I}=100\;m$, Δα=30°, LOS and NLOS conditions.

The presented results show the diversity of $p_{S}\left(\theta ,\phi \right)$ and $p_{I}\left(\theta ,\phi \right)$ both due to the relationship between the beam lobes and surface areas under the graphs that correspond to the received powers, $P_{S}$ and $P_{I}$, respectively. This fact indicates the dependence of the received power on the main lobe orientation of the gNodeB beam pattern in relation to the UE-I. This directly influences the determined SIR value. We observe the same situation in the DL scenario. A detailed analysis of the simulation test results is described in the next section.

## 5. Simulation Results

For the assumptions described in Section 4, we carried out simulation studies using the MATLAB environment. The results for the DL and UL scenarios are discussed in Section 5.1 and Section 5.2, respectively. Section 5.3 contains the comparison of inter-beam interference evaluation obtained based on the MPM and 3GPP statistical model [14]. In this case, we chose the DL scenario to present exemplary results.

### 5.1. DL Scenario

**Figure 6:**
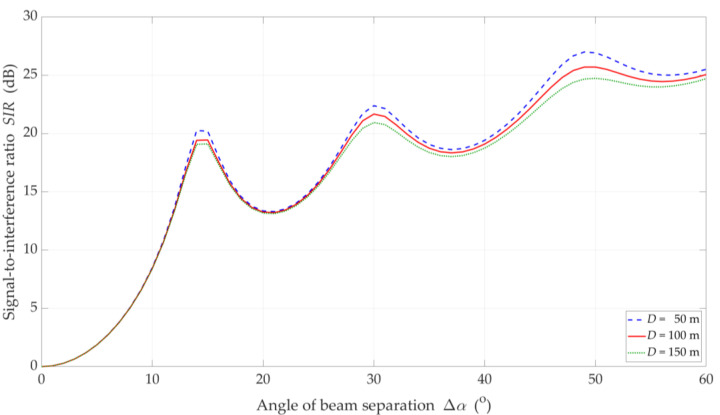
SIR versus separation angle for DL scenario, selected *D* = {50, 100, 150} m, and LOS (TDL-D) conditions.

**Figure 7:**
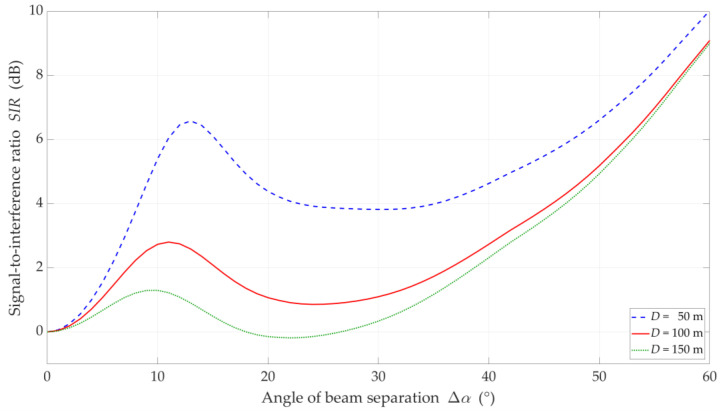
SIR versus separation angle for DL scenario, selected *D* = {50, 100, 150} m, and NLOS (TDL-B) conditions.

**Figure 8:**
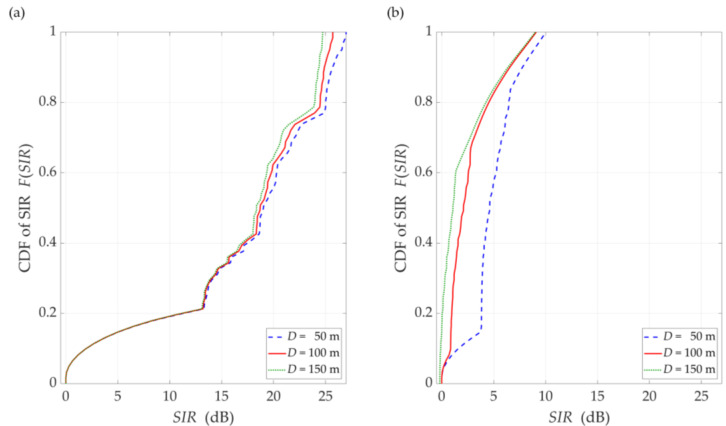
CDFs of SIR for DL scenario, selected *D* = {50, 100, 150} m, under (**a**) LOS (TDL-D) and (**b**) NLOS (TDL-B) conditions.

**Figure 9:**
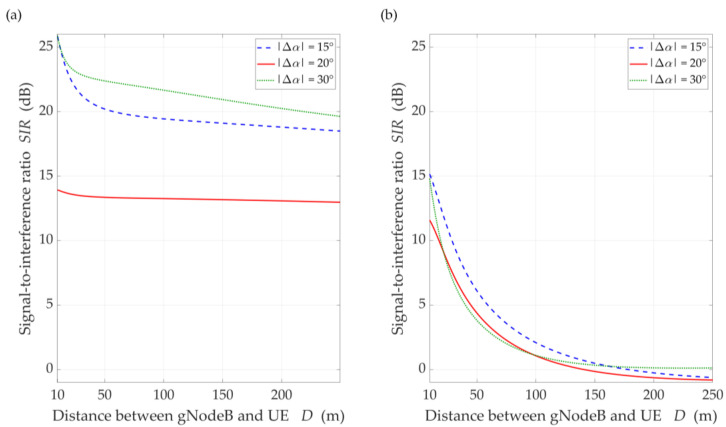
SIR versus distance for DL scenario, selected ∆*α* = {15°, 20°, 30°}, under (**a**) LOS (TDL-D) and (**b**) NLOS (TDL-B) conditions.

**Figure 10:**
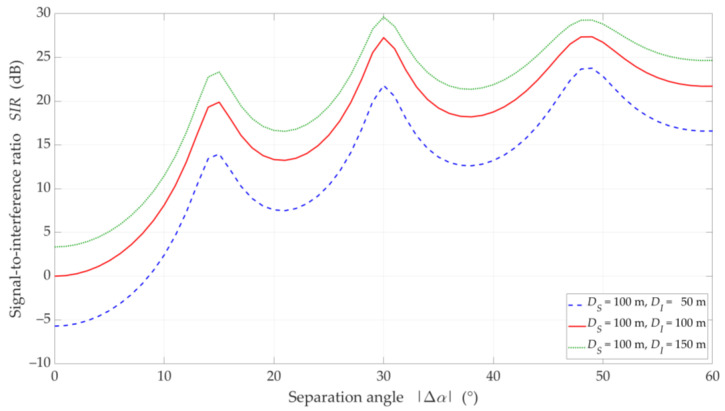
SIR versus separation angle for UL scenario, selected *D_I_* = {50, 100, 150} m, *D_S_* = 100 m, and LOS (TDL-D) conditions.

The simulation results for the DL scenario are presented in Figure 6, Figure 7, Figure 8, Figure 9 and Figure 10. Figure 6 and Figure 7 show the SIR graphs versus separation angles for selected distances between the gNodeB and UE, under LOS and NLOS conditions, respectively. Based on these charts, we also determined cumulative distribution functions (CDFs) of SIR, F(SIR), presented in Figure 8.

The increase in the separation angle reduces the downlink interference between the reference beam providing services to the UE and the interference beam. However, the nature of the SIR graphs is not uniform. For Δα={15°,30°,48°}, there are local maxima. We may observe this effect both for LOS and NLOS conditions. It results from considering side lobes in the realistic patterns of the base station beams. As the distance D increases, these maxima are less and less significant. We obtain the similar results in [32] for the carrier frequency of 3.5 GHz.

On the other hand, the obtained results differ significantly from those presented in [43], where some stabilization may be seen in the SIR graphs. In [43], two simplifications are assumed. Firstly, the Gaussian main lobe pattern without side lobes is modeling as the beam. Secondly, the beam gain is constant regardless of its radiation direction. Whereas, in the real beamforming antenna array, the beam gain depends on its direction. This second fact influences importantly on the differences in the presented results.

The comparison of the CDFs (see Figure 8) shows that for 80% of the results of the LOS simulation tests, we obtain up to 20 dB better beam separation compared to NLOS conditions. In the absence of a direct propagation path, we can observe an increase in the SIR value by 5 dB in over 80% of the results, whereas this increase is below 1 dB for LOS conditions. Figure 9 illustrates the SIR versus the gNodeB-UE distance for selected Δα, under LOS and NLOS conditions.

Analyzing the obtained results, we can see that as the distance increases, the SIR is reduced. The rationale for this effect is as follows. In the simulation study scenarios, we assume that the environment is homogeneous in terms of propagation properties. This means that the PDP is constant in all directions of electromagnetic wave emission. This assumption complies with the conditions of the propagation phenomena analysis described and recommended by 3GPP [14]. In relation to the model MPM, this means that an increase in the gNodeB-UE distance causes an apparent increase in large and a decrease in small semi-axes of all half-ellipsoids. As a result, the reception of the propagation paths which originate from the main lobe of the interfering beam is focused on the direction of maximum reception of the UE antenna. This causes an increase in the interference level relative to the power of the useful signal by about 7 dB. However, for Δα=20°, we see an evident influence of the side lobes on the increase of the interference level, which results in the reduction of the SIR to 13 dB in LOS simulations. The concentration of the interfering paths on the direction of maximum reception also occurs in the NLOS conditions. In this case, the uniformity of the spreading of all propagation paths lowers the range of SIR variation about 10 dB and reduces the differentiation of the side lobes’ influence.

For LOS conditions, we can also observe that, despite the larger separation angle for Δα=20°, we obtain a lower useful beam resistance to interference compared to Δα=15°. This effect is the result of the concentration of the received power on the side-lobe direction and the first minimum of the useful Rx beam, respectively. Figure 10 shows that this phenomenon does not occur under NLOS propagation conditions. The scattering phenomenon of electromagnetic waves under these conditions makes it impossible to concentrate the received power in the Tx-Rx direction.

### 5.2. UL Scenario

**Figure 11:**
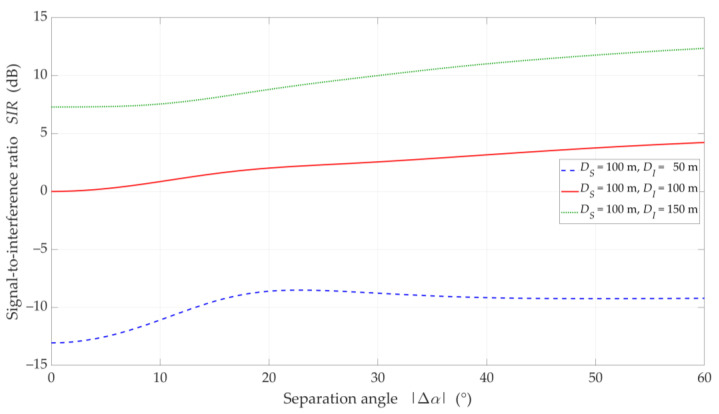
SIR versus separation angle for UL scenario, selected *D_I_* = {50, 100, 150} m, *D_S_* = 100 m, and NLOS (TDL-B) conditions.

**Figure 12:**
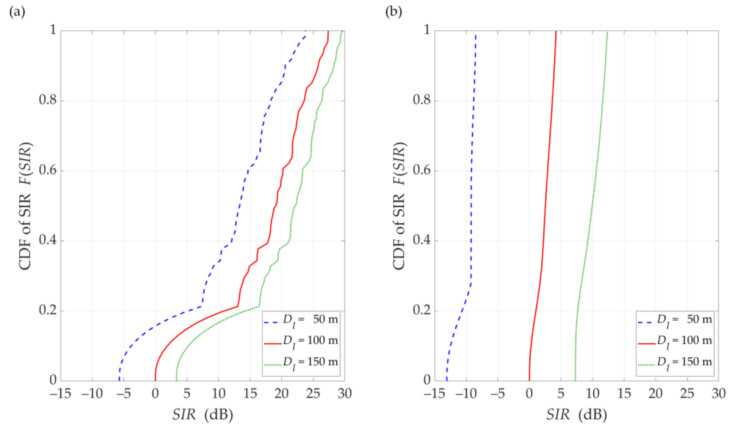
CDFs of SIR for UL scenario, selected *D_I_* = {50, 100, 150} m, *D_S_* = 100 m, (**a**) LOS (TDL-D) and (**b**) NLOS (TDL-B) conditions.

**Figure 13:**
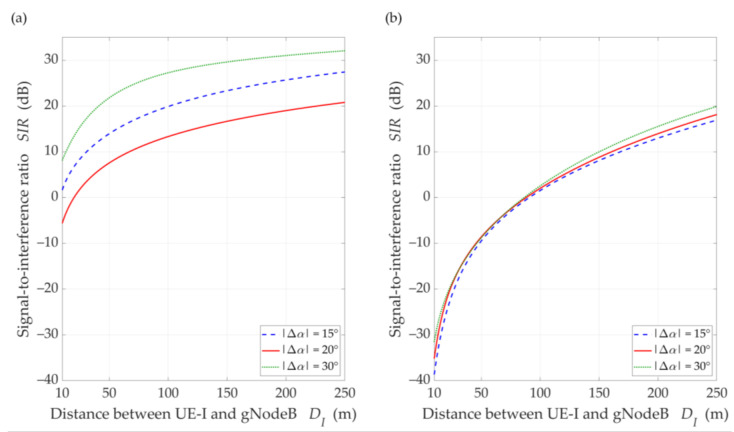
SIR versus distance *D_I_* for UL scenario, selected ∆*α* = {15°, 20°, 30°}, *D_S_* = 100 m, under (**a**) LOS (TDL-D) and (**b**) NLOS (TDL-B) conditions.

In Figure 10, Figure 11, Figure 12 and Figure 13, the simulation results are depicted for the UL scenario. Figure 10 and Figure 11 present the SIR curves versus separation angles for selected distances between the gNodeB and UE-I, under LOS and NLOS conditions, respectively. Figure 12 shows the CDFs of SIR for the UL scenario, which were obtained based on curves in Figure 10 and Figure 11.

The obtained results are evident because the SIR graphs correspond to the inversion of the gNodeB beam pattern for the useful link. This testifies the correctness of the developed simulation procedure. The comparison of graphs in Figure 10 and Figure 11 shows the smoothing effect of a multipath propagation environment on changes in the SIR as a function of Δα. Figure 11 shows that as the distance between the UE-I and gNodeB increases, the shape of the analyzed graph becomes similar as to the graph for LOS conditions. In this case, the distance increase contributes to the convergence of the signal reception directions to the distribution concentrated around the UE-I-gNodeB direction. Similar as to the DL scenario, the comparative analysis of the CDFs (see Figure 12) shows better beam separation with respect to the NLOS conditions. In this case, for 80% of the simulation test results, the SIR value may be about 25 dB greater. The graphs illustrated in Figure 10 and Figure 11 also show that under both LOS and NLOS conditions, to ensure the desired quality of the received signal, i.e., a given value of the SIR, the separation angle decreases with increasing the distance. From a practical viewpoint, this conclusion is obvious. However, the possibility of quantitative SIR assessment in multi-beam radio links operating under NLOS conditions determines the originality of the presented solution. This fact is a premise for the practical use of the developed method in the process of planning and power control in radio links with multi-beam antenna systems.

Figure 13 displays the SIR charts versus $D_{I}$ for $D_{S}=100\;m$, selected Δα, under LOS and NLOS conditions. The presented results are obtained for Δα equal to 15°, 20°, and 30°. For these values, the gNodeB beam pattern of the serving link reaches the first minimum, maximum of the first side-lobe, and second minimum, respectively (see Figure 10).

Analyzing the results for LOS conditions, we can see the same effect as for the DL simulation study scenario. Despite the larger separation angle for Δα=20°, we obtain a lower useful beam resistance to interference compared to Δα=15°. Of course, the reason for this effect is the same as in the DL scenario. For NLOS conditions, the scattering phenomenon of electromagnetic waves under these conditions makes it impossible to concentrate the received power in the Tx-Rx direction. Therefore, we do not see this effect in Figure 13b. The obtained results show the possibility of the SIR evaluation for various propagation conditions enabling optimal management of co-channel beams, which is the basis for interference mitigation, minimizing energy and spectral resources of wireless networks.

### 5.3. Exemplary Comparison of MPM with 3GPP Approach for DL Scenario

In this section, we provide an example comparison of the proposed MPM-based approach with another solution. In our opinion, choosing a different propagation model that can be the basis for a similar analysis of the inter-beam interference level is not easy. It results from the fact that only a few propagation models make it possible to consider the parameters and patterns of antennas and the environmental scattering of signals occurring in a radio channel. The statistical model based on the 3GPP standard [14] is one of them. Moreover, the choice of the 3GPP model was dictated by three reasons. Firstly, the analysis carried out in this paper with the use of the MPM is based on TDLs defined in the same standard [14]. Secondly, in both simulators we considered the same antenna patterns created according to the 3GPP recommendation [42]. Thirdly, we were able to use a proprietary simulator of the 3GPP statistical model, which was developed in the MATLAB environment and is used for generating the results contributed to the 3GPP, as an input to 5G standardization or research studies (e.g., [9,44]).

Exemplary interference comparison determined based on the MPM and 3GPP model was carried out for the DL scenario and the distance D=100 m. To obtain the average SIR, $SIR_{\mathrm{avg}}$, the Monte Carlo method with 1000 repetitions of statistical channel model realizations was used in the 3GPP simulator. Based on the set of obtained results, confidence intervals for $SIR_{\mathrm{avg}}$ with the standard deviation $\sigma_{SIR}$ were also determined. The same parameters as in Section 5.1 were adopted in the research.

**Figure 14:**
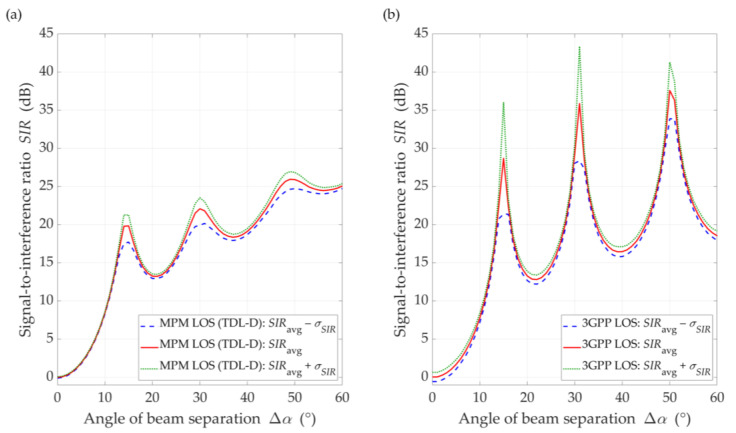
SIR comparison between (**a**) MPM and (**b**) 3GPP model for DL scenario, *D* = 100 m, and LOS conditions.

**Figure 15:**
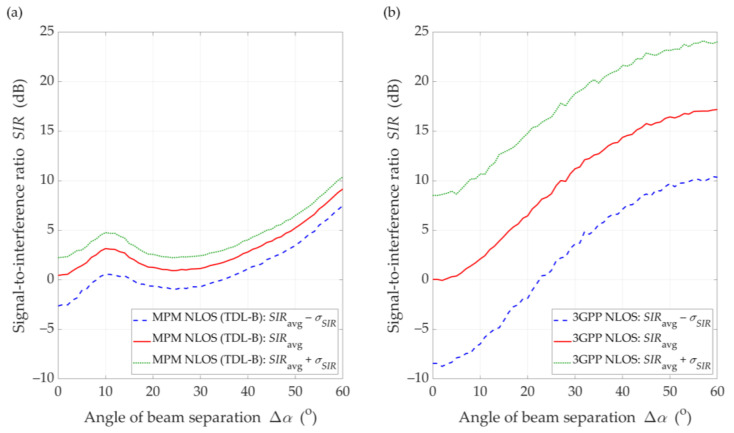
SIR comparison between (**a**) MPM and (**b**) 3GPP model for DL scenario, *D* = 100 m, and NLOS conditions.

To compare the MPM and 3GPP approaches, we ran the MPM simulator also in Monte Carlo mode for 1000 runs. Thus, the mean results, $SIR_{\mathrm{avg}}$, with the confidence intervals, $SIR_{\mathrm{avg}}\pm \sigma_{SIR}$, were determined. The results of the MPM and 3GPP comparison for the DL scenario and the distance D=100 m between the gNodeB and UE are illustrated in Figure 14 and Figure 15 for LOS and NLOS conditions, respectively.

Overall, we might conclude that the results are similar. The SIR results are more similar for LOS conditions (see Figure 14), where we may see characteristic extremes resulting from the use of the same gNodeB antenna pattern. In this case, both the maxima and the minima fall for the same separation angles. This is due to the presence of a direct path that enhances or reduces the influence of the pattern side lobes in certain directions relative to its main lobe (see Figure 4 and Figure 5). Therefore, the optimal directions’ selection for the adjacent beams in the gNodeB based on the MPM and 3GPP approaches will be identical or very similar. In this case, we would like to emphasize that the MPM approach gives the possibility to obtain an average result from a single simulation, while the 3GPP statistical model requires the time-consuming Monte Carlo methodology.

Under NLOS conditions (see Figure 15), the dynamics of SIR changes is lower than for LOS conditions. The results for the MPM and the 3GPP model show that the multipath propagation environment for NLOS conditions and the lack of a direct path provide to minimize the impact of the transmitting antenna pattern lobes. Thus, the selection of optimal directions for the adjacent gNodeB beams should be made based on an analysis for LOS conditions. On the other hand, we would like to highlight that other propagation models available in the literature also indicate result differences with the 3GPP model, e.g., [45,46,47,48].

With regard to the presented above comparative analysis, it is also worth providing the mean values of the standard deviation, ${\bar{\sigma }}_{LOS/NLOS}^{Model}$, obtained for the MPM: ${\bar{\sigma }}_{LOS}^{MPM}=0.58\;\mathrm{dB}$ and ${\bar{\sigma }}_{NLOS}^{MPM}=1.69\;\mathrm{dB}$, and 3GPP model: ${\bar{\sigma }}_{LOS}^{3GPP}=0.97\;\mathrm{dB}$ and ${\bar{\sigma }}_{NLOS}^{3GPP}=7.51\;\mathrm{dB}$ under LOS and NLOS conditions, respectively. The differentiation of the obtained results is related to the different spatial nature of scatterings (i.e., spatial dispersion) in the two analyzed propagation models. In the MPM, we use a multi-ellipsoidal geometric structure which is defined based on the TDL of the 3GPP standard [14]. On the other hand, the 3GPP statistical model has greater flexibility in the spatial location of potential scatterers. A more detailed comparison of the MPM and 3GPP model will be presented in the prepared next paper focusing on the 3.5 GHz band used in 5G massive-MIMO systems. A more detailed description of the 3GPP model and simulator will be presented there.

## 6. Conclusions

This paper is devoted to assessing the limitations that exist in multi-beam antenna systems. Here, the SIR is the primary metric that is used to evaluate the level of interference between the intra-cell beams. The presented procedure for assessing this parameter is based on the PAS analysis, which is determined by simulation tests. By using the channel transmission characteristics (i.e., TDL or PDP) to create the geometric MPM structure, the results of assessing the received power are closely related to the different propagation environment type. Using the MPM allows mapping the impact of the antenna beam radiation patterns on the PAS. The presented methodology allows evaluating changes in the PAS as a function of antenna beam shape and parameters such as the maximum radiation direction, main and side-lobes beamwidths. Additionally, the ability to evaluate the SIR under both LOS and NLOS conditions justifies using this method in the network planning process of energy and spectral management of 5G system with the multi-beam antenna systems. In the multipath propagation environment, in most cases to evaluate fluctuations in the received signal level, the Rician and Rayleigh distributions are used for LOS and NLOS conditions, respectively. The SIR assessment is the basis for determining the parameters of these characteristics. Due to the association of the SIR with the propagation properties of the environment, it is justified to use the presented SIR assessment methodology in the 5G network planning. The ability to adapt the developed model to any environment, weather conditions, and multi-beam antenna system distinguishes this SIR determination method from among the methods used so far. The comparison of the mean results for the proposed methodology with a similar approach based on the 3GPP statistical model shows that the same optimal directions for the adjacent gNodeB beams might be determined faster based on the MPM approach. A more detailed comparison of the two solutions with regard to the interference level assessment in the 3.5 GHz band will be presented in the authors’ next work [49]. In the future, we also plan to conduct empirical research for selected scenarios that will allow us to verify the approach presented in this paper.

## Data Availability

The data presented in this study are available on request from the corresponding author. The data are not publicly available due to project restrictions.

## Abbreviations

3D	three-dimensional
3GPP	3rd Generation Partnership Project
5G	fifth-generation
AOA	angle of arrival
AOD	angle of departure
CDF	cumulative distribution function
DL	downlink
GBM	geometry-based model
gNodeB	5G base station
ITU	International Telecommunication Union
LOS	line-of-sight
MIMO	multiple-input-multiple-output
mmWave	millimeter-wave
MPM	multi-ellipsoidal propagation model
NLOS	non-line-of-sight
NR	New Radio
PAS	power angular spectrum
PDP	power delay profile
Rx	receiver
SIR	signal-to-interference ratio
TDL	tapped delay line
Tx	transmitter
UE	user equipment
UE-I	interfering UE
UE-S	serving UE
UMa	urban macro
UL	uplink

## Symbols

(θ,ϕ)	AOA of individual propagation path
$\left(\theta_{T},\phi_{T}\right)$	AOD of individual propagation path
${\left|g\left(\theta ,\phi \right)\right|}^{2}$	normalized power pattern of receiving antenna
${\left|g_{I}\left(\theta_{T},\phi_{T}\right)\right|}^{2}$	normalized power pattern of interfering transmitting antenna
${\left|g_{S}\left(\theta_{T},\phi_{T}\right)\right|}^{2}$	normalized power pattern of serving transmitting antenna
$\alpha_{R}$	direction of receiving beam
$\alpha_{RI}$	direction of interfering receiving beam
$\alpha_{RS}$	direction of serving receiving beam
$\alpha_{T}$	direction of transmitting beam
$\alpha_{TI}$	direction of interfering transmitting beam
$\alpha_{TS}$	direction of serving transmitting beam
Δα	separation angle between serving and interference beams
ΔPL	path loss correction coefficient (relationship between attenuation of propagation environment for different distances)
$\gamma_{\theta }$	angular dispersion of local scattering components in elevation plane
$\gamma_{\phi }$	angular dispersion of local scattering components in azimuth plane
θ	elevation AOA of individual propagation path
$\theta_{T}$	elevation AOD of individual propagation path
$\Phi_{0}$	direction of receiving (gNodeB) beam in relation to direction of cell sector center in UL scenario
$\Phi_{I}$	direction of interfering transmitting (gNodeB) beam in relation to direction of cell sector center in DL scenario
$\Phi_{S}$	direction of serving transmitting (gNodeB)beam in relation to direction of cell sector center in DL scenario
ϕ	azimuth AOA of individual propagation path
$\phi_{T}$	azimuth AOD of individual propagation path
$\sigma_{SIR}$	standard deviation of SIR for confidence interval analysis
${\bar{\sigma }}_{LOS}^{3GPP}$	standard deviation of SIR for 3GPP model and LOS conditions
${\bar{\sigma }}_{NLOS}^{3GPP}$	standard deviation of SIR for 3GPP model and NLOS conditions
${\bar{\sigma }}_{LOS/NLOS}^{Model}$	standard deviation of SIR for Model and LOS/NLOS conditions
${\bar{\sigma }}_{LOS}^{MPM}$	standard deviation of SIR for MPM and LOS conditions
${\bar{\sigma }}_{NLOS}^{MPM}$	standard deviation of SIR for MPM and NLOS conditions
$\tau_{n}$	delay of *n*th time-cluster in PDP/TDL
a	auxiliary variable used to compute $r_{T}$
$a_{xn}$	major semi-axis of *n*th ellipsoid along *x*-axis
$b_{yn}$	minor semi-axis of *n*th ellipsoid along *y*-axis
$C_{0}$	normalizing constant
c	lightspeed
$c_{zn}$	minor semi-axis of *n*th ellipsoid along *z*-axis
D	distance between Tx and Rx or between gNodeB (Rx) and UE (Tx) in DL
$D_{I}$	distance between UE-I (Tx) and gNodeB (Rx) in UL
$D_{S}$	distance between UE-S (Tx) and gNodeB (Rx) in UL
F(SIR)	CDF of SIR
$f\left(\tilde{p}\right)$	distribution of path power
$f_{0}\left(\theta ,\phi \right)$	2D von Mises distribution describing local scattering components
$f_{I}\left(\theta_{T},\phi_{T}\right)$	distribution of AOD for interfering link
$f_{S}\left(\theta_{T},\phi_{T}\right)$	distribution of AOD for serving link
G	gain of receiving beam
$G_{I}$	gain of interfering transmitting beam
$G_{S}$	gain of serving transmitting beam
$I_{0}\left(\cdot \right)$	zero-order modified Bessel function of imaginary argument
N	number of all time-clusters in analyzed PDP/TDL
n	number of analyzed time-cluster in PDP/TDL
$P_{I}$	power of interfering signal
$P_{S}$	power of serving signal
PL	path loss
$PL\left(D_{I}\right)$	path loss for wireless links between UE-I and gNodeB at distance $D_{I}$
$PL\left(D_{S}\right)$	path loss for wireless links between UE-S and gNodeB at distance $D_{S}$
$\tilde{p}$	power of individual propagation path
$p_{n}$	mean power of *n*th time-cluster in PDP/TDL (*n*th local extreme of PDP/TDL)
$p_{I}\left(\theta ,\phi \right)$	PAS seen at the output of receiving antenna for interfering link
$p_{S}\left(\theta ,\phi \right)$	PAS seen at the output of receiving antenna for serving link
${\tilde{p}}_{I}\left(\theta ,\phi \right)$	PAS in vicinity of receiving antenna for interfering link
${\tilde{p}}_{S}\left(\theta ,\phi \right)$	PAS in vicinity of receiving antenna for serving link
$r_{T}$	radial coordinate in spherical system with origin in Tx
SIR	SIR
$SIR_{\mathrm{avg}}$	average SIR for confidence interval analysis
$SIR_{\mathrm{avg}}\pm \sigma_{SIR}$	confidence intervals of SIR
